# A novel kinase inhibitor, Regorafenib, blocks EGFR-dependent signaling to repress tumour metastasis in human triple-negative breast cancers

**DOI:** 10.3389/fcell.2026.1714597

**Published:** 2026-03-03

**Authors:** Ludong Zhao, Chunxin Wang, Yanzhi Wang, Meijing Liu, Hongyu Du, Xiaohui Chu, Wenqiang Yang, Wei Zhao, Guan Wang, Bo Zhang, Yue Zhang, Yajing Meng, Hongdan Li, Pushuai Wen, Ying Liu

**Affiliations:** 1 Liaoning Technology and Engineering Center for Tumor Immunology and Molecular Theranotics, Collaborative Innovation Center for Age-related Disease, Life Science Institute of Jinzhou Medical University, Jinzhou, Liaoning, China; 2 College of Basic Medical Sciences, Jinzhou Medical University, Jinzhou, Liaoning, China; 3 The First Affiliated Hospital, Jinzhou Medical University, Jinzhou, Liaoning, China; 4 Department of Obstetrics and Gynaecology, LKS Faculty of Medicine, University of Hong Kong, Hong Kong, Hong Kong SAR, China

**Keywords:** EGFR, EGFR-STAT3, Regorafenib, Src-JNK/p38-YAP1 signaling, triple-negative breast cancer

## Abstract

**Introduction:**

Triple-negative breast cancer (TNBC), the most aggressive subtype, poses a significant challenge as approved targeted therapies are lacking. The epidermal growth factor receptor (EGFR) is highly expressed in over 50% of TNBC cases and is implicated as a key driver in TNBC progression. Regorafenib, a small-molecule inhibitor of multiple receptor tyrosine kinases, is utilized as a second-line treatment for metastatic tumors.

**Methods:**

Bioinformatics analysis and clinical analysis of 14 breast cancer cases revealed the EGFR expression and activation in tumor tissues. Functional validation through *in vitro* and *in vivo* models demonstrated EGFR’s oncogenic role and Regorofenib**’**s inhibitory effect. Mechanistically, multi-molecular biology methods and transcriptomics analysis identified EGFR-associated pathway driving TNBC lung metastasis.

**Results:**

In this study, we demonstrate that Regorafenib, acting as an inhibitor of EGFR, exhibits potent anti-metastatic effects both *in vitro* in TNBC cell lines expressing EGFR and *in vivo* in mouse models of TNBC lung metastasis. Mechanistically, Regorafenib-mediated EGFR inhibition suppresses signaling in the Src-JNK/p38-YAP1 pathway, decreases STAT3 and NF-κB activation, thereby impeding epithelial-to-mesenchymal transition and metastasis.

**Discussion:**

Our discovery of Regorafenib as a novel inhibitor of EGFR activation provides valuable insights for TNBC-specific anti-EGFR therapies targeting metastasis.

## 1 Introduction

Breast cancer, the prevalent malignant tumor in women globally, is a primary cause of cancer-related fatalities in this demographic (Sun et al., 2025; Yan et al., 2025; Liu K. et al., 2024). It exhibits notable invasiveness and metastatic capacity, disseminating to nearby lymph nodes through the lymphatic system and to remote organs such as the lungs, bones, liver, and brain via the circulatory system (Park et al., 2022). This characteristic is a primary factor contributing to its unfavorable prognosis. Consequently, elucidating the molecular mechanisms underlying breast cancer metastasis and devising early intervention strategies have emerged as critical scientific priorities for enhancing patient clinical outcomes.

Breast cancer is categorized into four subtypes: luminal A, luminal B, human epidermal growth factor receptor 2 (HER2)-positive, and TNBC (Thakur et al., 2024). Research has demonstrated that HER2 is excessively expressed in approximately 20%–25% of breast cancer instances. This overexpression is closely linked to high tumor invasiveness (Ferrando-Díez et al., 2022). This type of breast cancer shares partial pathological and molecular features with basal-like breast cancer (BLBC)- a tumor subtype defined by distinct cytokine expression signatures and a non-luminal, basal-type molecular genetic profile (Parker et al., 2023; Perou et al., 2000). The majority of basal-like breast tumors are TNBC, the most aggressive subtype in breast cancer categorizations. TNBC, comprising only 15%–20% of cases, frequently appears in advanced stages, resulting in elevated recurrence rates, worse patient outlooks, and reduced survival probabilities (Garrido-Castro et al., 2019). Moreover, TNBC lacks targeted therapies, and its cancer cells easily spread to distant sites like the brain, bones, lungs, and liver (Li S. et al., 2022).

Epithelial-mesenchymal transition (EMT) is vital in starting breast cancer metastasis (Malla et al., 2022). EMT and its reverse process, namely mesenchymal-epithelial transition (MET), are widely present throughout the entire process of wound healing, fibrosis, and tumor progression. EMT is closely related to the malignancy degree of tumors, including tumor invasion, metastasis, and drug resistance (Glaviano et al., 2025; Lüönd et al., 2021). Matrix metalloproteinases (MMPs) can cleave extracellular matrix (ECM) proteins. This creates conditions for tumor cells to enter and exit blood circulation (endothelial and extravascular leakage), accelerating tumor cell metastatic dissemination (Chen X. et al., 2023; Huang et al., 2022). Hence, inhibiting MMP expression and reversing EMT could effectively hinder the metastatic capability of TNBC cells, offering novel insights for TNBC treatment.

EGFR is overexpressed or activated in over 50% of TNBC and BLBC patients. Its abnormal expression correlates significantly with larger tumor size, distant metastasis, and poor prognosis (Bi et al., 2023; Subham et al., 2023). EGFR is involved in cell migration, EMT, cell attachment, invasion, and breakdown of ECM primarily through the PI3K/Akt, Ras/Raf/MEK/ERK, and STAT3 routes (Bi et al., 2023; Liu J. et al., 2024). In TNBC, BLBC, and inflammatory breast cancer, targeting the EGFR signaling pathway has become essential.

Epidermal growth factor receptor (EGFR, also known as ErbB1, HER1) is a typical representative of the ErbB/HER family of receptor tyrosine kinases (RTKs). When lacking ligands, EGFR exists in a monomeric state, with its kinase domain in an autoinhibited state. Binding of ligands like epidermal growth factor (EGF) triggers the formation of homodimers or heterodimers among EGFR and its family members ErbB2/HER2, ErbB3/HER3, and ErbB4/HER4, thereby inducing conformational changes in the kinase domain and activating it (Ferguson, 2008; Jura et al., 2009; Rybak et al., 2023). At the same time, during this process, the kinase domain may also be activated by EGFR dimerization reactions induced by local concentration increases (Rybak et al., 2023). EGFR, as a member of the receptor tyrosine kinase (RTK) family, was initially applied in ligand binding-related studies. The research has clearly demonstrated the significance of ligand-induced dimerization (Sabbah et al., 2020). This diversity of regulation is partly achieved through the combination of multiple ligands with EGFR, or through the formation of heterodimers of EGFR and other ErbB family receptors (Blanco and Kang, 2011).

Regorafenib, a TKI taken by mouth, is authorized for managing mCRC that is resistant, GISTs that are advanced after prior imatinib and sunitinib treatment, and HCC that is inoperable following sorafenib therapy (Grothey et al., 2020). Preclinical studies indicate Regorafenib targets multiple kinases. Involved in tumor angiogenesis are vascular endothelial growth factor receptors (VEGFRs), tyrosine kinase with immunoglobulin-like and EGF-like domains 2 (TIE2), fibroblast growth factor receptors (FGFRs), and platelet-derived growth factor receptors (PDGFRs). Also implicated in proliferation is KIT, RAF, and rearranged during transfection (RET). Furthermore, they play a role in the tumor microenvironment and metastasis (VEGFR2-3, PDGFR) (Schmieder et al., 2014; Wilhelm et al., 2011; Zopf et al., 2016). Currently, research on Regorafenib in breast cancer, particularly on its mechanism of action, remains scarce.

Our findings indicate that EGFR is upregulated in ER^+^, HER2^+^, and TNBC, and is associated with poor prognosis. Moreover, we explored the relationship between EGFR overexpression/activation and EMT. Furthermore, using *in vitro* models and *in vivo* TNBC lung-metastasis models, we evaluated Regorafenib’s inhibitory effect on TNBC metastasis. Additionally, we preliminarily investigated EGFR-regulated signaling pathways involved in TNBC lung metastasis.

## 2 Materials and methods

### 2.1 Reagents and antibodies

Regorafenib (HY-10331), HGF (HY-P7121), BAY 60-6583 (HY-103171), Dehydroepiandrosteron (HY-100197), TGF-β1 (HY-p70543) were purchased from MedChemExpress (Monmouth Junction, NJ, USA). TSFSILMSPDSPD peptides were synthesized by Beijing Qingke Biological Company. EGFR (51071-2-AP), HER2 (18299-1-AP), vimentin (10366-1-AP), N-cadherin (22018-1-AP), E-cadherin (20874-1-AP), p-STAT3 (ET1603-40), p-JNK (80024-1-RR), β-tubulin (66031-1-Ig) primary antibody were purchased from Proteintech Biological Company (Wuhan, China). p-EGFR (3777S), Trop2 (47866S), p-Src (2105S), p-p38 (4511T), PKM2 (4053T) primary antibody were purchased from Cell Signaling Technology (Beverly, United States). MMP1 (YT2792) and p-YAP1 (R26121) primary antibody were purchased from Immunoway Technology (Texas, United States). EGF (GF316) and LPA (L-7260) were purchased from sigma Biotechnology (CA, United States).

### 2.2 Patient samples

Human lung tumor tissues were obtained from the Department of Breast Surgery at the First Affiliated Hospital of Jinzhou Medical University. Total protein was extracted from the freshly collected breast tumor tissues for Western blot analysis. The study protocol received approval from the Medical Ethical Committee of the First Affiliated Hospital of Jinzhou Medical University (permit number: JZMULL2025538), and written informed consent was provided by all patients.

### 2.3 Vector construction

The plasmids of pCDH-EGFR-GFP-puro and pCMV-Flag-EGFR-del19 + L858R were purchased from Wuhan Miaoling Biological Technology Company.

### 2.4 Cell culture, transfection and lentivirus infection

The human breast cell lines MDA-MB-468, JIMT-1, and SK-BR-3 were purchased from Punosai Biotechnology Company (Wuhan, China), the MCF-7 and 293T cell lines in-house. All cell lines were maintained in Dulbecco’s modified Eagle’s medium containing 10% FBS, and100 U/mL penicillin-streptomycin at 37 °C under 5% CO_2_ incubator. Periodic detection of *mycoplasma* contamination with a commercial kit (Beyotime, Beijing, China) was negative. After precultured in DMEM with 10% FBS medium for 24 h, cells were washed once with PBS and starved overnight in FBS-free DMEM, followed by treatment of the indicated concentration and duration of EGF treatments. For transient transfection polyethylenimine (PEI) method was employed with plasmid-PEI mixture (3:1 w/w ratio) being added to cells and medium replaced 6 h post transfection. Lentiviral Infection: lentivirus was produced from pLEX-131G using the ViraPower Lentiviral Expression System (Invitrogen).

### 2.5 Glo-kinase assay

Luciferase-based Glo-kinase assay for quantitative measurement of kinase activity in the presence of residual ATP. The kinases use up ATP in the substrate phosphorylation reaction other hand luciferase produces light considering the concentration of ATP (Fatma and Siddique, 2025). The assay was performed according to the Kinase-Glo luminescent kinase assay protocol (Promega, V6711). The quick and dirty of it was that reactions were established in white microplates by combining substrate, kinase (or buffer for control samples) and ATP to kick off the reaction, with a final volume of ∼100 µL per well. The plate was incubated at 37 °C for periods of 30 min which allow time for the kinase to consume free ATP within the well. Then 50 μL of detection reagent was added to an equal volume and incubated at room temperature for 10–30 min to allow residual ATP to react with luciferase generating a stable luminescence. Luminescence was measured using a luminometer, and kinase activity of the sample quantified by comparing the amount of remaining ATP with that of a pyruvate standard curve.

### 2.6 Cell viability assay

Cell viability was determined by employing the CCK-8 kit (Beyotime, Beijing, China). To each well, 10 µL of CCK-8 solution was added, and the cells were then incubated for 1 h. Following this, spectrophotometric scanning of the plates was conducted at 450 nm.

### 2.7 Transwell and migration assay

MDA-MB-231 cells were treated with Regorafenib and cultured until 90% confluence in 6-well plates. Scratch was made in a pellicle with a cellular monolayer using a 10 μL tip and observed using microscopy.

Then the upper chamber was plated with 5.0 × 10^4^ of serum-free medium containing MDA-MB-231 cells and the lower chamber with complete medium to culture at 37 °C in a humid atmosphere containing 5% CO_2_ for 24 h. Twenty-four hours after incubation, the inserts were taken out, fixed in 4% paraformaldehyde for 30 min, and then stained with crystal violet for 15 min.

### 2.8 Immunohistochemistry (IHC)

Human breast cancer tissue microarray (TMA) slides containing 74 breast cancer samples and 8 adjacent non-tumor tissue samples were used for IHC analysis. The staining intensity was graded from 0 to 3, and the percentage of positive cells was categorized from 0 to 4. Final IHC scores, ranging from 0 to 12, were calculated by multiplying the intensity score (0–3) by the percentage of positive cells score (0–4). Cases were classified into low or high groups based on the median IHC score (Criswell et al., 2022). The relationship between EGFR expression levels and clinicopathological parameters in breast cancer patients was then evaluated.

### 2.9 Mouse tumor model and treatment regimens

Female NOD-SCID mice (6–8 weeks old, 20–22 g) were procured from Beijing Weitong Lihua Laboratory Animal Co., Ltd., China. These animals were housed in specific pathogen-free facilities at Jinzhou Medical University’s Laboratory Animal Center. The study received ethical approval from Jinzhou Medical University’s Animal Experimentation Ethics Committee (Registration No. 240209-5).

A mouse model of breast cancer lung metastasis was induced by injecting 5 × 10^5^ MDA-MB-231 cells via the tail vein. Subsequently, the mice were randomly assigned to either the model group or the treatment group. Following 4 weeks of Regorafenib treatment, the mice were euthanized, and the lungs were excised to quantify metastatic nodules.

Our laboratory established the MMTV-PyMT mouse model of spontaneous breast cancer lung metastasis. Transgenic PyMT (MMTV-PyMT) mice were obtained from The Jackson (JAX) Laboratory (stock no: 002374) and bred on FVB/n and PyMT (MMTV-PyMT) backgrounds (Fu et al., 2022). These mice developed spontaneous breast cancer after 2 months. Subsequently, they were randomly allocated to the model group or the treatment group. After 4 weeks of Regorafenib treatment, the mice were euthanized, and the number of metastatic nodules in the lungs was assessed. All experiments utilized littermates from control and transgenic mice.

### 2.10 RNA isolation, cDNA synthesis, and quantitative real-time polymerase chain reaction (qRT-PCR)

Isolation of total RNA, synthesis of cDNA, and conducting quantitative real-time RT-PCR (qRT-PCR) were carried out following the protocol outlined in (Li Y. et al., 2022). The primer sequences can be found in Supplementary Table S1 of the Supplementary Data. Gene expression levels were determined using the 2^−ΔΔCT^ method, with 18S rRNA serving as the internal control.

### 2.11 RNA sequencing (RNA-seq)

RNA was isolated from breast cancer lung metastases and adjacent tissues using Trizol reagent. RNA quality and quantity were assessed with a NanoDrop 200 spectrophotometer, and integrity was evaluated using an Agilent 210 Bioanalyzer. Libraries were prepared with the VAHTS Universal V6 RNA-seq Library Prep Kit. Transcriptome analysis was conducted by OE Biotech Co., Ltd., with bioinformatics analysis in R Studio. Principal component analysis was performed to check for sample duplication. Hierarchical cluster analysis of differentially expressed genes was carried out to visualize gene expression patterns. A radar map of the top 30 genes showing expression levels of DEGs was created using the gradar package in R. Gene Set Enrichment Analysis was conducted to evaluate predefined gene sets and determine enrichment levels based on differential gene expression.

### 2.12 Protein extraction and western blotting

Whole-cell or tissue extracts were prepared in ice-cold lysate buffer (50 mM NaF, 1 mM Na_3_VO_4,_ 1 mM DTT, 1 mM PMSF, with proteinase and phosphatase inhibitors cocktail). Total protein concentration was determined using the Pierce BCA Protein Assay Kit following the manufacturer’s guidelines (Thermo Fisher Scientific, United States). Proteins (20–30 μg) were separated by 10% SDS-PAGE electrophoresis and transferred to polyvinylidene difluoride (PVDF) membranes. For non-reducing gradient gel electrophoresis, protein samples were prepared with native gel loading buffer (P0016N, Beyotime, Shanghai, China). Subsequently, the protein lysates were subjected to polyacrylamide gel electrophoresis and transferred to PVDF membranes. The membranes were blocked in 5% non-fat milk in TBS-T buffer for 1 h and then incubated overnight at 4 °C with the specified primary antibodies. Afterward, the polyvinylidene difluoride membranes were incubated with appropriate secondary antibodies for 1 h at room temperature. Membranes were visualized using the ECL kit (Thermo Fisher Scientific, Waltham, MA, United States) and Image Quant LAS400 imaging system (GE Healthcare Life Sciences, Chicago, IL, United States).

### 2.13 Fluorescence microscopy assays

MCF-7 cells expressing EGFR or EGFR-del19 + L858R were cultured on glass coverslips, followed by triple PBS washes, fixation with 4% paraformaldehyde, and permeabilization with 0.25% Triton X-100. Vimentin or N-cadherin was immunostained using specific antibodies and Alexa Fluor-conjugated secondary antibodies. Imaging was performed with Olympus 1120 laser-scanning confocal microscope and Olympus 550 software.

Tumors and lung tissues were fixed in 4% paraformaldehyde, paraffin-embedded, sectioned at 3 μm, and stained with hematoxylin and eosin (H&E) following standard procedures. Imaging was performed with Leica Aperio Versa 200 and Leica DM4B microscopes.

### 2.14 Statistical analysis

All data were presented as mean ± standard deviation (SD), with statistical significance set at P < 0.05, otherwise denoted as nonsignificant (n.s). Animal studies involved age-matched and randomly assigned subjects based on body weight. Parametric data were assessed using an unpaired Student’s t-test (for comparing two group means) or standard one-way analysis of variance (ANOVA) (for comparing mean differences among more than 2 groups). Statistical analyses were conducted using GraphPad Prism 8.0 software (San Diego, CA, United States).

## 3 Results

### 3.1 Analysis of EGFR gene amplification and overexpression in human breast cancer tissues

**FIGURE 1:**
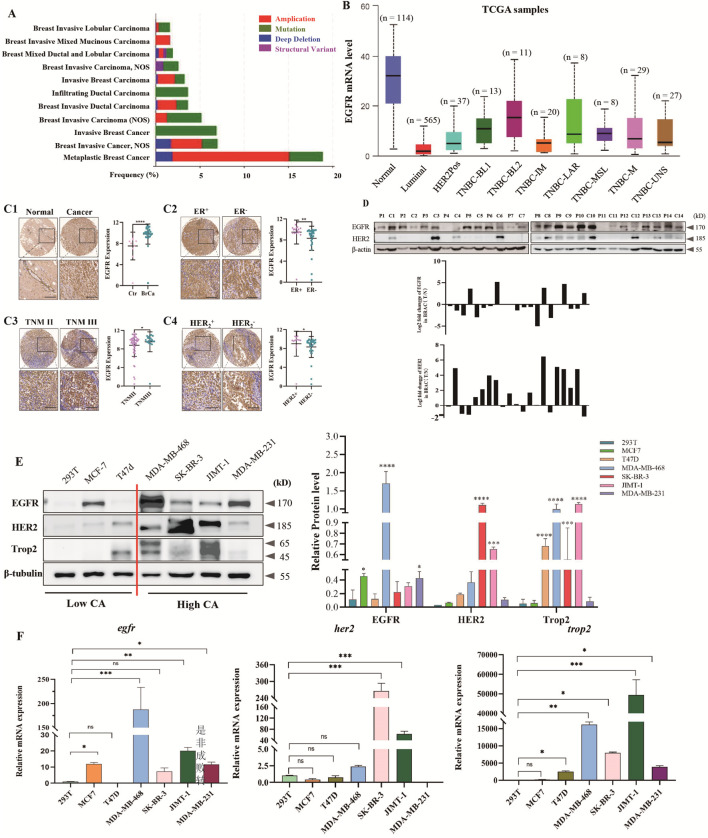
EGFR is highly expressed and hyperactivated in breast cancer, which is associated with poor prognosis. **(A)** Analysis of EGFR amplification and mutation frequencies (shown as percentages) in various types of breast cancer from the TCGA database. **(B)** EGFR mRNA expression levels in luminal breast cancer, HER2-positive breast cancer, triple-negative breast cancer (TNBC), and normal controls from the TCGA database (http://ualcan.path.uab.edu/cgi-bin/ualcan-res.pl). **(C)** Immunohistochemical (IHC) analysis of EGFR expression using tissue microarrays. **(C1)** Representative images and quantification of EGFR immunohistochemical staining in breast cancer (n = 74) and non-tumor breast tissues (Ctrl) (n = 8). Scale bar for all images = 50 μm. **(C2)** Representative images and quantification of EGFR immunohistochemical staining in ER-positive breast cancer (n = 17) and ER-negative breast cancer (n = 30). Scale bar for all images = 50 μm. **(C3)** Representative images and quantification of EGFR immunohistochemical staining in TNM stage II (n = 51) and TNM stage III (n = 19) breast cancer tissues. **(C4)** Representative images and quantification of EGFR immunohistochemical staining in HER2-negative (n = 32) and HER2-positive (n = 11) breast cancer tissues. Scale bar for all images = 50 μm. All data are presented as mean ± SD. Statistical analysis was performed using one-way ANOVA; *P < 0.05, **P < 0.01, ****P < 0.0001, ns: not significant. **(D)** Western blot analysis of EGFR or HER2 relative expression in 14 breast cancer tissues (C) and paired adjacent non-tumor tissues (P) (Cohort II). The Y-axis represents the log2-transformed fold change of EGFR C/P protein expression ratio. **(E)** Immunoblotting analysis and quantification of EGFR, HER2, or Trop2 protein expression in normal cells (293T), ER-positive breast cancer cells (MCF7 and T47D), HER2-positive breast cancer cells (JIMT-1 and SK-BR-3), and TNBC cells (MDA-MB-468 and MDA-MB-231). All data are presented as mean ± SD. Statistical analysis was performed using one-way ANOVA; *P < 0.05, ***P < 0.001, ****P < 0.0001 vs. the 293T group. **(F)** qPCR analysis of *egfr*, *her2*, or *trop2* mRNA levels in 293T cells and different breast cancer cell lines. All data are presented as mean ± SD. Statistical analysis was performed using one-way ANOVA; *P < 0.05, **P < 0.01, ***P < 0.001, ns: not significant.

Based on the analysis results of the Cbioportal (https://www.cbioportal.org/) cancer genome database, it was shown that the copy number of EGFR frequently changed in various types of breast cancer, comprising invasive breast cancer, including mixed ductal and lobular carcinoma, invasive ductal carcinoma, metaplastic breast cancer, invasive lobular carcinoma, invasive mixed mucinous carcinoma, and invasive breast cancer (NOS), and metaplastic breast cancer (Figure 1A). In particular, there was a high frequency of gene amplification (more than 10%) in patients with metaplastic breast cancer. Moreover, TCGA dataset analysis of EGFR mRNA levels revealed significantly upregulated EGFR gene expression in invasive breast cancer tissues relative to adjacent normal tissues. Meanwhile, compared with all types of breast cancer, the EGFR gene had the highest expression in the basal-like-2 (BL-2) subtype of TNBC (Figure 1B). At the same time, the expression of EGFR protein was analyzed by EGFR immunohistochemistry using breast cancer tissue microarrays. The findings indicated that the expression level of EGFR protein in cancerous tissues was notably higher than that in adjacent normal tissues (Figure 1C1). Furthermore, compared with ER^−^-or HER2^-^-breast cancer tissues, the expression levels of EGFR protein in ER^+^- breast cancer tissues or HER2^+^-breast cancer tissues were significantly increased (Figures 1C2, C4). In addition, the expression level of EGFR increased with the upgrading of the TNM stage of breast cancer (Figure 1C3).

Western blot analysis detected EGFR and HER2 protein levels in 14 breast cancer and adjacent normal tissue samples. Results indicated high EGFR expression in the majority of breast cancer tissues, while the HER2 protein was only highly expressed in some tissue samples, which may be related to the types of tumor samples selected (Figure 1D). Western blot and RT-qPCR were employed to assess EGFR and HER2 levels in breast cancer cells. Findings revealed markedly elevated EGFR levels in the highly invasive High CA cells compared to the less invasive Low CA cells. Additionally, EGFR expression was notably higher in the triple-negative MDA-MB-468 and MDA-MB-231 cells than in the HER2+ SK-BR-3 and JIMT-1 cells. In addition, the tumor-associated calcium signal transducer 2 (Trop2), a specific molecular marker for breast cancer invasion and migration, was also highly expressed in the highly invasive breast cancer cell line (Figure 1E). The mRNA expression results of *egfr*, *her2*, and *trop2* were basically consistent with the protein expression results (Figure 1F).

### 3.2 Activation or overexpression of EGFR promotes EMT, invasion, and migration of breast cancer epithelial cells

**FIGURE 2:**
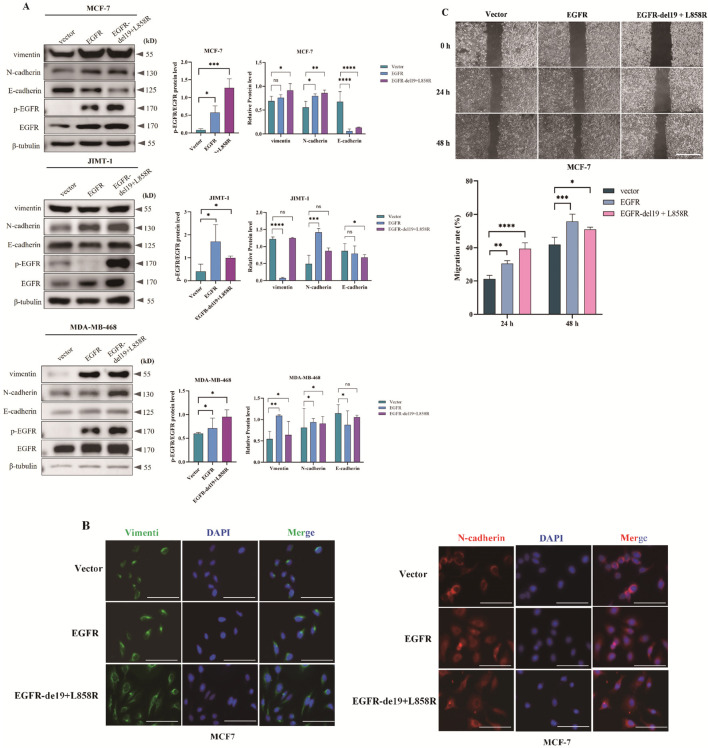
EGFR activation and overexpression promote EMT transformation, migration, and invasion in breast cancer cells. The 19 exon deletion and L858R mutation are classic EGFR-activating mutations, which enable EGFR protein to undergo constitutive phosphorylation and activation without ligand stimulation, accounting for 80%–90% of EGFR mutations. In epithelial-like breast cancer cell lines (MCF-7, JIMT-1, and MDA-MB-468), stable EGFR-activating mutants and EGFR-overexpressing cell lines were screened. **(A)** Western blot analysis of characteristic molecular markers during EMT. The results of grayscale analysis for protein expression are presented in the figure. All data are presented as mean ± SD. Statistical analysis was performed using one-way ANOVA; *P < 0.05, **P < 0.01, ***P < 0.001, ****P < 0.0001, ns: not significant. **(B)** Immunofluorescence observation of EMT-specific molecules (vimentin and N-cadherin) localization. **(C)** Wound-healing assay to evaluate cell migration and invasion ability. All data are presented as mean ± SD. Statistical analysis was performed using one-way ANOVA; *P < 0.05, **P < 0.01, ***P < 0.001, ****P < 0.0001.

EMT is capable of enhancing the survival, migration, and invasion of tumor cells, which in turn facilitates the progression of malignant tumors in breast cancer (Rybak et al., 2023). Therefore, we constructed an EGFR self-activating vector plasmid: pCMV-EGFR-del19 + L858R. This plasmid contains a deletion of exon 19 of the EGFR receptor and a mutation at the L858 lysine site, enabling the activation of the EGFR receptor without the addition of ligands (EGF). In addition, we constructed an EGFR overexpression vector plasmid and screened stable-expressing cell lines. Furthermore, Western blot analysis was conducted to evaluate the impact of EGFR activation or overexpression on EMT markers in breast cancer epithelial cells MCF7, JIMT-1, and MDA-MB-468. The results showed that EGFR activation or overexpression significantly upregulated the expression of EMT markers vimentin and N-cadherin, while inhibiting the expression of E-cadherin (Figure 2A). In MCF-7 cells, overexpression of EGFR and EGFR-del19 + L858R significantly upregulated Vimentin expression by ∼1.1-fold and ∼1.3-fold, respectively, and upregulated N-cadherin expression by ∼1.4-fold and ∼1.5-fold, respectively, while E-cadherin expression downregulated by ∼8.9% and ∼19.7%, respectively. In JIMT-1 cells, overexpression of EGFR and EGFR-del19 + L858R significantly upregulated Vimentin expression by ∼0.9-fold and ∼1.0-fold, respectively, and upregulated N-cadherin expression by ∼2.8-fold and ∼1.8-fold, respectively, while E-cadherin expression downregulated by ∼10% and ∼22%, respectively. In MDA-MB-468 cells, overexpression of EGFR and EGFR-del19 + L858R significantly upregulated Vimentin expression by ∼2.0-fold and ∼1.2-fold, respectively, and upregulated N-cadherin expression by ∼1.2-fold and ∼1.1-fold, respectively, while E-cadherin expression downregulated by ∼24% and ∼10%, respectively. Similarly, the results of detecting the expression of EMT-related genes by RT-qPCR showed that EGFR activation or overexpression could significantly increase the expression of *vimentin*, *n-cadherin* and decrease the expression of *e-cadherin* (Supplementary Figure S1).

To further validate the functional role of EGFR in the observed phenotypes, we performed both pharmacological inhibition and genetic loss-of-function studies. Cells were treated with Regorafenib, a multi-kinase inhibitor targeting EGFR and other kinases, or transfected with siRNA specifically targeting EGFR. Consistent with the results from Regorafenib treatment, EGFR knockdown significantly suppressed migration and EMT process compared with non-targeting control siRNA (Supplementary Figures S3A,B). These data demonstrate that the effects of Regorafenib are at least partially mediated through inhibition of EGFR signaling, and confirm a critical role for EGFR in tumor cell migration and progression in this model.

A potential limitation of pharmacological inhibitors is their risk of off-target effects. To mitigate this concern and reinforce our conclusions, this study combined pharmacological inhibition with Regorafenib and targeted EGFR knockdown through RNA interference. The consistent phenotypic changes observed with both methods provide strong evidence that the effects are specifically mediated by EGFR, rather than by non-specific actions of Regorafenib. This dual validation strategy enhances the causal relationship between EGFR signaling and tumor cell migration in our model system.

Immunofluorescence detected vimentin and N-cadherin localization in MCF-7 cells, revealing that EGFR activation or overexpression increased their expression significantly (Figure 2B). This suggests that EGFR activation or overexpression can trigger EMT in breast cancer cells. Furthermore, the scratch assay demonstrated that EGFR activation or overexpression notably boosted MCF-7 cell migration potential (Figure 2C).

In conclusion, breast cancer cells of epithelial origin may induce EGFR activation or overexpression, thereby triggering EMT, invasion, and migration.

### 3.3 Regorafenib, a small-molecule inhibitor of EGFR, effectively suppresses the development of breast cancer

**FIGURE 3:**
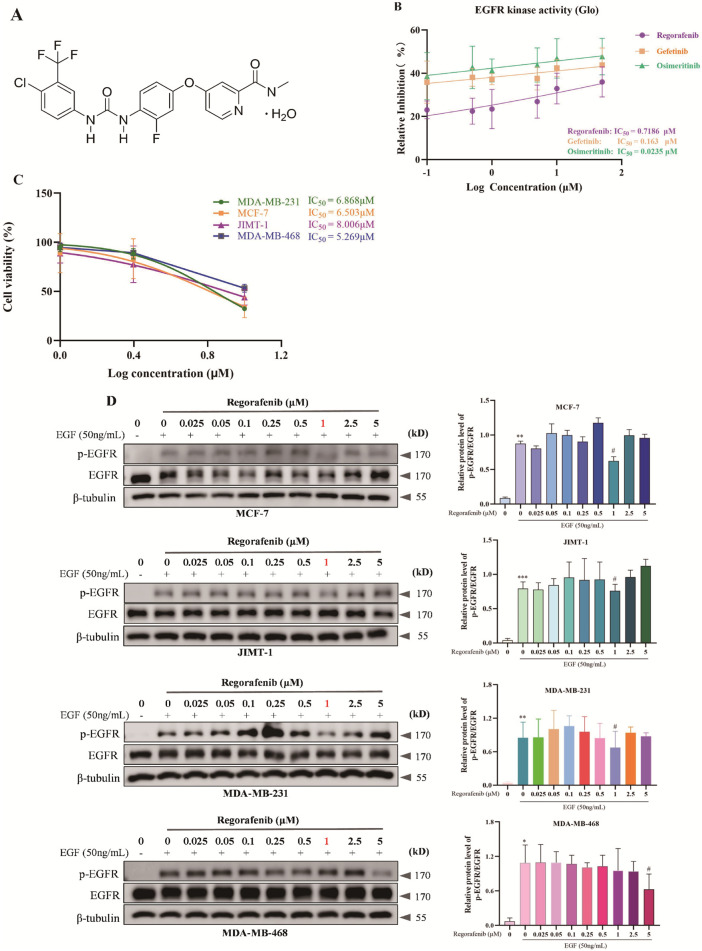
Regorafenib significantly inhibits EGFR kinase activity. **(A)** Chemical structure of Regorafenib. **(B)** Regorafenib-mediated inhibition of EGFR kinase activity was evaluated using the Glo-kinase assay system *in vitro*. The half-maximal inhibitory concentration (IC_50_) was subsequently calculated. **(C)** Breast cancer cells were treated with indicated concentrations of Regorafenib for 24 h, and cell proliferation was measured by CCK-8 assay. Based on the inhibition profile, the IC_50_ of Regorafenib against breast cancer cells was calculated. Data are presented as mean ± SD of three independent experiments. Statistical analysis was performed using one-way ANOVA, with group comparisons by t-test. **(D)** Breast cancer cells were serum-starved overnight, treated with indicated concentrations of Regorafenib for 24 h, and stimulated with 50 ng/mL EGF for 5 min before cell lysis. Total protein was extracted, and EGFR phosphorylation levels were analyzed by Western blot. The results of grayscale analysis for protein expression are presented in the figure. All data are presented as mean ± SD. Statistical analysis was performed using one-way ANOVA; *P < 0.05, **P < 0.01, ***P < 0.001, vs. control group, and #*P* < 0.05, vs. EGF group.

Given the crucial role of EGFR activation in EMT, invasion, and migration of breast cancer, we introduced Regorafenib, a small-molecule inhibitor of EGFR, to explore its function in the development of breast cancer. The chemical structure of Regorafenib is shown in Figure 3A. We evaluated the inhibitory activity of EGFR kinase using the Glo-kinase method. Meanwhile, we also assessed the effects of established EGFR inhibitors, Gefitinib and Osimertinib, on EGFR kinase. The calculated IC_50_ values indicated that the Regorafenib treatment group had an IC_50_ value of 0.7186 μM. In contrast, the Gefitinib treatment group exhibited an IC_50_ value of 0.163 μM, while the Osimertinib treatment group demonstrated an IC_50_ value of 0.023 μM. Notably, the IC_50_ value for Regorafenib was comparable to those of Gefitinib and Osimertinib (Figure 3B). Subsequently, the CCK-8 assay was employed to determine the impact of Regorafenib on the proliferative viability of breast cancer cells. The findings demonstrated that Regorafenib exerted an inhibitory effect on the proliferation of breast cancer cells in a dose-dependent fashion. The IC_50_ values were 5.269 ± 1.521 μM for MDA-MB-468 cells, 6.503 ± 1.043 μM for MCF7 cells, 6.868 ± 0.248 μM for MDA-MB-231 cells, and 8.006 ± 0.878 μM for JIMT-1 cells (Figure 3C).

Furthermore, to ensure the rationality of the experimental concentrations in this assay, we carefully considered the well-characterized inhibitory effect of Regorafenib on EGFR kinase activity and its subsequent impact on breast cancer cell survival. Based on the results of Glo-kinase activity analysis and the CCK-8 assay, we conducted a concentration gradient screening experiment to determine the optimal working concentration of Regorafenib. Our data indicated that the breast cancer cells were exposed to varying Regorafenib concentrations, and Western blot analysis revealed a notable suppression of EGFR activation. Our data indicated that Regorafenib had the most significant inhibitory effect at 1 μM (Figure 3D). Therefore, we selected 1 μM as the treatment concentration for all subsequent experiments.

### 3.4 Regorafenib inhibits lung metastasis of breast cancer

**FIGURE 4:**
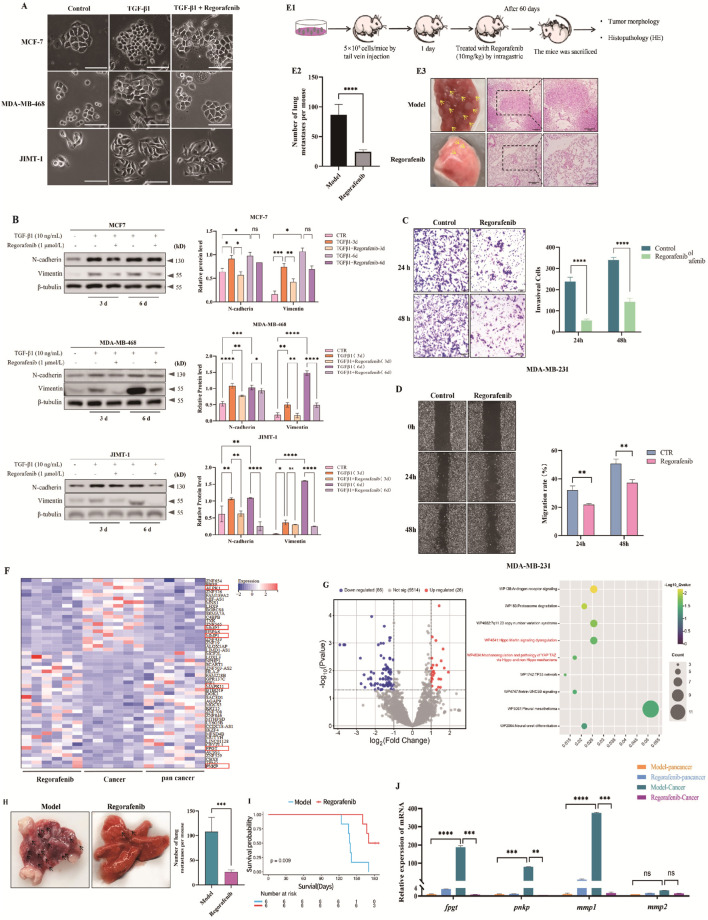
Regorafenib inhibits EMT and metastasis processes induced by EGFR receptor activation or overexpression in human breast cancer. **(A)** Phase-contrast microscopy showing morphological changes in MCF-7, MDA-MB-468, and JIMT-1 cells after 6 days of treatment with TGF-β1 alone or TGF-β1 combined with Regorafenib. **(B)** Western blot analysis of EMT marker protein expression in MCF-7, MDA-MB-468, and JIMT-1 cells treated with TGF-β1 alone or TGF-β1 combined with Regorafenib for different durations. **(C)** Transwell assay assessing the invasive ability of MDA-MB-231 cells after Regorafenib treatment for different durations. The invasion percentage was calculated using ImageJ software, with statistical analysis performed by one-way ANOVA and t-test for intergroup comparisons. The results of grayscale analysis for protein expression are presented in the figure. All data are presented as mean ± SD. Statistical analysis was performed using one-way ANOVA; *P < 0.05, **P < 0.01, ***P < 0.001, and ****P < 0.0001. **(D)** Wound-healing assay evaluating the migratory ability of MDA-MB-231 cells after Regorafenib treatment for different durations. The wound closure percentage was analyzed using ImageJ software, with statistical analysis performed by one-way ANOVA and t-test for intergroup comparisons. All data are presented as mean ± SD. Statistical analysis was performed using one-way ANOVA; **P < 0.01. **(E)** Regorafenib inhibits lung metastasis in an MDA-MB-231 tail vein injection mouse model. **(E1)**: Experimental flowchart: MDA-MB-231 cells (5 × 10^5^/200 μL/mouse) were injected into the tail vein of SCID mice. Regorafenib (10 mg/kg) was administered via oral gavage every other day for 8 weeks before sacrifice (n = 6 per group). And the lung nodule formation was assessed. **(E2)**: Statistical analysis of lung nodule counts, with one-way ANOVA and t-test for intergroup comparisons, ****P < 0.0001. **(E3)**: Images of lung metastatic foci (left, arrows indicate nodules) and H&E staining of lung nodules (right). **(F)** Heatmap analysis of transcriptome sequencing results from lung nodules (tumor) and matched distal normal lung tissues (adjacent normal). The color scale represents the log2-transformed FPKM/TPM values (red, upregulated; blue, downregulated), with rows indicating genes and columns representing individual samples. **(G)** Volcano plot of differentially expressed genes (DEGs) and KEGG pathway enrichment analysis for lung nodule tumor and matched distal normal lung tissues. For the KEGG enrichment panel: X-axis represents the proportion of pathway-associated genes in all DEGs (gene ratio); Y-axis indicates the significantly enriched KEGG pathways. For the volcano plot panel: X-axis denotes log2 (fold change) of DEGs, Y-axis represents -log10 (P value), with red and blue dots indicating significantly upregulated and downregulated DEGs, respectively (|log2(fold change)| ≥ 1, P < 0.05). **(H)** Regorafenib inhibits spontaneous breast cancer lung metastasis in MMTV-PyMT mice. Macroscopic lung images (left, arrows indicate nodules) and statistical analysis of nodule counts (right). **(I)** Kaplan-Meier survival curves of model mice in different experimental groups (n = 6 per group). Survival data were statistically analyzed using the log-rank test for intergroup differences in survival outcomes; one-way ANOVA and unpaired t-test were applied for comparison of other relevant indicators among groups. **(J)** Q-PCR analysis of invasion- and metastasis-related genes from transcriptome sequencing in lung nodules (tumor) and distal normal lung tissue (adjacent normal) of MMTV-PyMT spontaneous breast cancer model mice. Data are presented as mean ± SD of triplicate experiments. Statistical analysis was performed by one-way ANOVA and t-test for intergroup comparisons. **P < 0.01, ***P < 0.001, and ****P < 0.0001.

To explore Regorafenib’s inhibition of EMT in breast cancer cells, we induced EMT in the cells using transforming growth factor TGF-β1, known to induce EMT (Cao et al., 2025; Zhang et al., 2023). We used epithelial-derived breast cancer cell lines: MCF-7, MDA-MB-468, and JIMT-1 for this part of the experiment. Initially, the cellular morphology was examined using an inverted phase-contrast microscope. It was found that compared with the control, after 3 days of TGF-β1 stimulation, the three cell lines of MCF-7, MDA-MB-468, and JIMT-1 all showed different degrees of mesenchymal-like cell morphological changes, manifested as irregular cell shapes, long spindle-shaped morphology, and multiple slender protrusions (Figure 4A). Among them, the morphological change of JIMT-1 cells was the most obvious, and that of MCF-7 cells was the least obvious. Meanwhile, the addition of Regorafenib could significantly inhibit the effect of TGF-β1 (Figure 4A). Following this, the expression of EMT-associated proteins was determined via western blot technique. The results showed that after 3 days of TGF-β1 stimulation, the epithelial - like cell marker (E-cadherin) was significantly downregulated, and the mesenchymal cell markers (N-cadherin, Vimentin) were all upregulated. The addition of Regorafenib could significantly inhibit the effect of TGF-β1 (Figure 4B). After 6 days of TGF-β1 stimulation, the epithelial-like cell marker E-cadherin was downregulated, while the mesenchymal-like cell markers N-cadherin and Vimentin were upregulated. The addition of Regorafenib could significantly inhibit the effect of TGF-β1, and the result was stronger than that after 3 days of TGF-β1 stimulation (Figure 4B). Among the three cell lines, JIMT-1 cells exhibited the most significant changes in EMT markers before and after treatment, while MCF-7 cells showed the least significant changes. These results were consistent with morphological observations.

TNBC, known for its aggressive nature, is associated with a high incidence of invasion and spread, leading to mortality in affected individuals. Understanding the mechanisms underlying TNBC metastasis necessitates a thorough investigation of the migratory and invasive traits of TNBC cells. Our study commenced by assessing the impact of Regorafenib on the migratory and invasive capacities of MDA-MB-231 TNBC cells. Initially, a scratch assay was conducted to assess the influence of Regorafenib treatment on TNBC cell migration. The findings depicted in Figure 4C reveal a notable reduction in cell migration rate upon Regorafenib addition compared to the control group. Furthermore, a Transwell invasion assay was employed to evaluate TNBC cell invasiveness. As illustrated in Figure 4D, Regorafenib treatment (1 μM) significantly attenuated the invasive potential of MDA-MB-231 TNBC cells relative to the control group.

To further confirm the specificity of Regorafenib in targeting EGFR signaling and to assess whether constitutively active EGFR can circumvent its inhibitory effects, we conducted rescue experiments by stably expressing EGFR (Del19 + L858R) in MCF-7 or MDA-MB-231 cells (Supplementary Figure S4). Assays following Regorafenib treatment revealed that, unlike wild-type EGFR-expressing cells, which showed significant inhibition of cell migration and the EMT, EGFR (Del19 + L858R)-expressing cells displayed marked resistance to Regorafenib. Specifically, the migration rate (Supplementary Figures S4A,B) and EMT process (Supplementary Figures S4C,D) in EGFR (Del19 + L858R)-expressing cells were not significantly altered by Regorafenib treatment. Additionally, we introduced another EGFR activation mutation, T790M, in these experiments, yielding results consistent with those observed for EGFR (Del19 + L858R). These findings clearly demonstrate that constitutively active EGFR (Del19 + L858R) or EGFR (T790M) can bypass the inhibitory effects of Regorafenib in epithelial cells.

In addition, we constructed a mouse model of breast cancer lung metastasis to analyze the effect of Regorafenib on inhibiting tumor metastasis. The experimental procedure is shown in Figure 4E1. Consistent with the *in vitro* experimental results, compared with the model group, the number of lung nodules in the mice of the Regorafenib treatment group was significantly reduced (Figure 4E2). Then, to confirm the effect of Regorafenib on TNBC lung metastasis, lung tissue was taken for H&E staining (Figure 4E3). The results showed that the model group showed obvious alveolar collapse, alveolar wall thickening, and tumor cell and inflammatory cell distribution; while the Regorafenib treatment group showed significant improvement in alveolar wall uniformity, alveolar thickening, and tumor cell and inflammatory cell infiltration, indicating that Regorafenib significantly inhibited breast cancer lung metastasis.

Furthermore, pulmonary metastatic lesions and adjacent tissues were separated for transcriptome sequencing. Six samples were tested in each group, and the samples were divided into three groups: cancer (pulmonary metastatic lesions in the model group), Regorafenib (pulmonary metastatic lesions in the Regorafenib treatment group), and pan cancer (tissues distal to pulmonary metastatic lesions in the Regorafenib treatment group). edgeR was used to analyze differentially expressed genes among samples. Figure 4F shows the differentially expressed genes in the three groups. In the heatmap, the upper part represents the genes significantly downregulated after Regorafenib treatment, while the lower part represents the genes significantly upregulated after Regorafenib treatment. The genes marked with red boxes are closely related to invasion and metastasis, including matrix metallopeptidase 1 (MMP1), matrix metallopeptidase 2 (MMP2), Mitogen-Activated Protein Kinase (MAPK); carbohydrate metabolism, such as Fucose-1-phosphate guanylyltransferase (FPGT); and NF-κB signaling, such as Alpha-kinase 1 (ALPK1), and they are the focus of our subsequent research. Subsequently, KEGG pathway enrichment analysis was performed on the differentially expressed genes among the three samples. Figure 4G shows the top 9 signaling pathways enriched by KEGG. These differential genes are mainly enriched in the Hippo-YAP1 signaling pathway. Results indicated that EGFR activation significantly upregulates the expression of Src and its downstream target YAP1, as well as the transcriptional targets of YAP1 that are known to promote metastasis. These signaling pathways are of particular interest to us, and we plan to validate them through cellular experiments in subsequent studies.

We employed the MMTV-PyMT mouse model of spontaneous breast cancer lung metastasis to assess Regorafenib’s impact on inhibiting tumor metastasis *in vivo*. Figure 4H displays the tumor cell count and pulmonary nodule count. The mice treated with Regorafenib exhibited a notable decrease in pulmonary nodules compared to the control group (P < 0.0001), demonstrating its efficacy in inhibiting breast cancer lung metastasis. The survival rate of mice in the Regorafenib group significantly surpassed that of the control group, as indicated by the growth curve (Figure 4I).

Furthermore, we extracted the total RNA from the lung tissues of the mice in the MMTV-PyMT spontaneous breast cancer lung metastasis model group and the Regorafenib group, and verified the expression of the differentially expressed genes screened in the transcriptome sequencing through RT-qPCR. The results are shown in Figure 4J. Compared with the model group, the RNA levels of Fucose-1-phosphate guanylyltransferase (*fpgt*), Polynucleotide kinase 3′-phosphatase (*pnkp*), and *mmp1* in the Regorafenib group were significantly downregulated, while there was no significant change in the gene expression of *mmp2*. These genes provide ideas for our subsequent research on breast cancer. In the follow-up, we will start from the above three genes to study their relevant relationships in breast cancer.

In conclusion, Regorafenib can inhibit the lung metastasis of TNBC and prolong the overall survival of the mouse model.

### 3.5 Regorafenib inhibits lung metastasis of breast cancer through suppressing the Src-JNK/p38 signaling pathway, reducing the phosphorylation of STAT3 and NF-κB, and increasing the phosphorylation of YAP1

**FIGURE 5:**
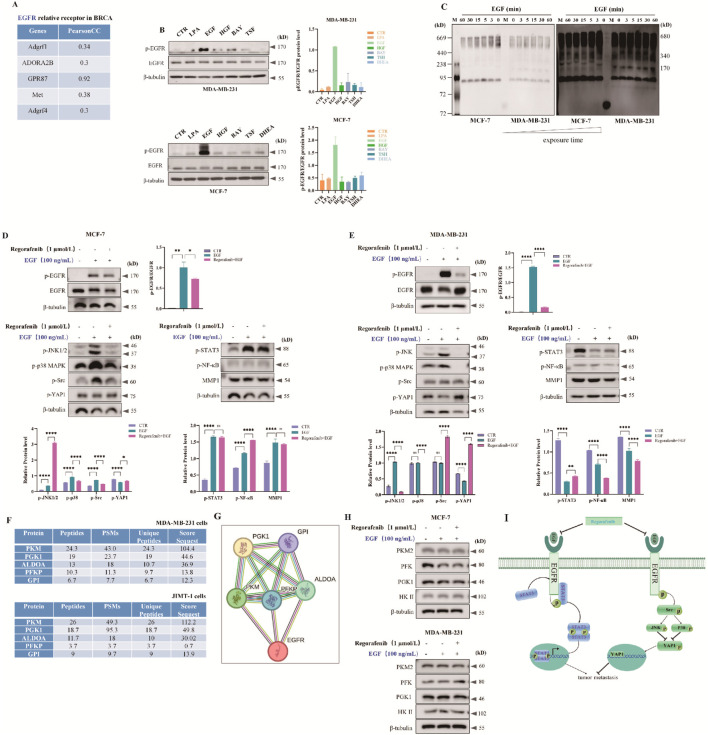
Regorafenib suppress EGFR-Src-JNK/p38-YAP1 and EGFR-STAT3 activation in human breast cancers. **(A)** Bioinformatics analysis of EGFR-related cell membrane receptors revealed correlations among the receptors as indicated by Pearson correlation coefficient (CC) values. **(B)** MDA-MB-231 or MCF-7 cells were treated with six EGFR-related receptor agonists, and EGFR activation was assessed via Western blot analysis. Cells were cultured in 6-well plates, subjected to overnight serum starvation, stimulated with various agonists for 5 min, and samples were collected. The specific agonists used were LPA (GPR87 receptor agonist, 200 μM), EGF (EGFR receptor agonist, 100 ng/mL), BAY (ADORA2B receptor agonist, 200 nM), TSF (Adgrf1 receptor agonist, 200 nM), and DHEA (Adgrf4 receptor agonist, 200 nM). The results of grayscale analysis for protein expression are presented in the figure. All data are presented as mean ± SD. **(C)** EGFR receptor monomer and multimer activation in MDA-MB-231 or MCF-7 cells were detected using native gel electrophoresis following stimulation with EGF (100 ng/mL) for varying durations. Total protein extracted from cell lysates was analyzed by PAGE gel electrophoresis with loading buffer devoid of SDS and β-mercaptoethanol to eliminate SDS interference in gel preparation and electrophoresis buffer. **(D,E)** Regorafenib was found to inhibit EGFR activation in both MCF-7 and MDA-MB-231 cells, consequently impeding downstream signaling pathway activation. MCF-7 cells **(D)** or MDA-MB-231 cells **(E)** were exposed to EGF alone or in combination with Regorafenib, and Western blot analysis was performed to evaluate the expression of downstream signaling pathway-associated proteins. **(F)** Endogenous co-immunoprecipitation (Co-IP) binding mass spectrometry (MS) was employed to identify proteins binding to EGFR in MDA-MB-231 or JIMT-1 cells. The enzymatic profiles relevant to glucose metabolism were assessed. **(G)** The String software was utilized to examine the relationship between EGFR and the aforementioned key enzymes involved in glucose metabolism. **(H)** Following this, MDA-MB-231 and MCF-7 cells were exposed to EGF alone or in combination with Regorafenib. Western blot analysis was conducted to evaluate the levels of key glucose metabolism enzymes. The experimental procedure involved culturing the cells in 6-well plates, depriving them of fetal bovine serum (FBS) after an overnight incubation, treating them with Regorafenib for 24 h, stimulating EGF-treated cells for 5 min, and extracting total protein for analysis of key enzymes in glucose metabolism via Western blot. Data are presented as mean ± SD of triplicate experiments. **(I)** A schematic model of EGFR-Src-JNK/p38-YAP1 signaling or EGFR/STAT3 signaling. Source data are provided as a Source Data file.

Through gene correlation analysis, we separately analyzed the genes related to EGFR in breast cancer. The results showed that the genes related to EGFR were mainly concentrated in the G protein-coupled receptor (GPCR) family and the receptor tyrosine kinase (RTK) family (Figure 5A). To detect the correlation between these receptors and the EGFR, six agonists were used: LPA (agonist of G Protein-Coupled Receptor 87), EGF (agonist of EGFR receptor), HGF (agonist of Hepatocyte Growth Factor Receptor), BAY (agonist of Adenosine A2b Receptor), TSF (agonist of Adhesion G Protein-Coupled Receptor F1), and DHEA (agonist of Adhesion G Protein-Coupled Receptor F4). These agonists were applied to stimulate MDA-MB-231 and MCF-7 cells respectively to observe the activation of corresponding receptors. In triple-negative breast cancer MDA-MB-231 cells, the EGFR receptor could be activated only by its own ligand EGF, similar results were observed in MCF-7 cells (Figure 5B). Furthermore, Western blot experiments were conducted to determine the optimal stimulation concentrations and durations of EGF. Results showed that in three breast cancer cell lines: MDA-MB-231, MCF-7, and JIMT-1, EGF stimulation exhibited a dose-dependent response. The EGFR was significantly activated when the EGF concentration reached 100 ng/mL, and maximal activation was achieved at 200 ng/mL. For subsequent experiments, 100 ng/mL of EGF was selected. In these three cell lines, EGF stimulation did not show a time-dependent pattern. The EGFR was notably activated after 5 min of stimulation, and activation essentially peaked after 15 min. Therefore, 5 min was chosen as the stimulation time for subsequent experiments (Supplementary Figure S2).

EGFR, a member of the RTKs situated on the cell membrane surface, mediates cellular processes including proliferation, differentiation, migration, and survival. They serve as both receptors and enzymes, binding ligands and triggering tyrosine residue phosphorylation in target proteins (Freed et al., 2017). Under normal circumstances, the ligands of RTKs bind to the extracellular domain to induce receptor dimerization and autophosphorylation of tyrosine residues in the cytoplasmic domain, thereby activating the receptors (Cho, 2020).

Accordingly, we initially investigated the dimerization and polymerization of EGFR. Native gel electrophoresis was employed to detect EGFR polymerization. As shown in Figure 5C, EGF stimulation could significantly induce the formation of EGFR dimers or multimers, with a remarkably small number of monomers. The above results indicate that the EGFR receptor activates through dimerization or polymerization to complete the signal transduction process.

Research has revealed that the MAPK pathway constitutes a signaling pathway specifically mediated by RTKs. Upon activation, RTKs can rapidly induce the phosphorylation of the adapter protein SHC, the formation of the SHC-Grb2-Sos complex, and the activation of MAPK. Therefore, we employed Western blot technology to detect the expression of proteins in the MAPK family after treatment with Regorafenib. In MCF-7 cells, Regorafenib effectively suppressed the activation of p38/MAPK and JNK/MAPK pathways triggered by EGF stimulation, with the participation of the non-receptor tyrosine kinase Src (Figure 5D). Furthermore, we investigated the activation of the Hippo-YAP1 pathway, which is crucial for processes such as tumor cell invasion, metastasis, and tissue development. The results demonstrated that EGF could distinctly inhibit the activation of YAP1, while Regorafenib obviously reversed the effect of EGF administration (Figure 5D). Furthermore, we investigated the phosphorylation of STAT3 and NF-κB, which are pivotal in diverse biological processes including inflammation, immune response, cell proliferation, and apoptosis. The results indicated that Regorafenib could significantly suppress the phosphorylation of STAT3 and NF-κB induced by the addition of EGF (Figure 5D). Furthermore, Regorafenib failed to notably inhibit the expression of extracellular matrix MMP1. This indicates that YAP1, STAT3 or NF-κB may be implicated in the invasion and metastasis processes of breast cancer, whereas the MMP1 might not be involved in these processes. However, this conclusion requires further verification and exploration in subsequent studies. Similarly, the results obtained in MDA-MB-231 cells were consistent with those in MCF-7 cells (Figure 5E). Thus, Regorafenib inhibited the metastasis of breast cancer through suppressing Src-JNK/p38 pathway, subsequently increased Hippo-YAP1 activation. We examined YAP1 expression in both the cytoplasm and nucleus to monitor its translocation between these two subcellular compartments. Our results demonstrated that in cell lines MCF-7 or MDA-MB-231, EGF treatment enhanced YAP1 translocation from the cytoplasm to the nucleus, whereas treatment with Regorafenib exerted an inhibitory effect on this process (Supplementary Figure S3). Meanwhile, Regorafenib could decrease STAT3 and NF-κB phosphorylation and inhibited the metastasis of breast cancer (Figures 5D,E).

Endogenous co-immunoprecipitation (Co-IP) followed by mass spectrometry (MS) in MDA-MB-231 and JIMT-1 cells identified EGFR interacting proteins. It was found that the levels of the enzymes on the glucose metabolic pathway such as PKM2,PGK1,PFKP, GPI, ALDOA appeared to be otherwise upregulated, losing to suggested that the pathological involvement of EGFR overexpression in the glucose metabolism (Figure 5F). In addition, a computational software was applied to predict the binding between EGFR and the above mentioned glucose-metabolism-related key enzymes, and evidence was found with the most relevant enzymes (Figure 5G), indicating that EGFR had interactions with these key enzymes. Western blot showed that regorafenib suppressed EGF-induced PKM2 activation but had no obvious influence on the other pivotal enzymes (Figure 5H). The specific mechanism remains to be further studied.

## 4 Discussion

Breast cancer, the most common malignant tumor in women globally, is a primary cause of cancer-related deaths in this population (Sun et al., 2025; Yan et al., 2025). Metastasis of tumors continues to be the primary factor leading to treatment ineffectiveness and mortality in cancer sufferers (Malla et al., 2022). As a locally originating malignant neoplasm, breast cancer exhibits substantial invasive and metastatic capabilities. It may spread to regional lymph nodes through the lymphatic system or metastasize to distant organs such as the lungs, bones, liver, and brain via the bloodstream. This multi-stage metastatic process both complicates the disease and severely limits the efficacy of existing treatment strategies (Thakur et al., 2024). Therefore, understanding the molecular mechanisms of breast cancer metastasis and developing early-intervention strategies are crucial scientific challenges for improving patient outcomes.

EGFR, the first identified receptor in the ErbB family of tyrosine kinases, is one member of this family that also includes ErbB2/Her2, ErbB3, and ErbB4 (Zou et al., 2023). In tumor cells, EGFR is abnormally activated through mechanisms such as gene amplification and transcriptional upregulation. Abnormal EGFR activation in tumor cells occurs via gene amplification and transcriptional upregulation. Increased EGFR protein and transcription levels are linked to unfavorable outcomes in different epithelial malignancies, such as colorectal cancer (CRC) (Zhu et al., 2021), non-small cell lung cancer (NSCLC) (Chen G. et al., 2023), endometrial cancer (Wang et al., 2023), and squamous cell carcinoma of the head and neck (SCCHN) (Wang et al., 2023). Moreover, in cancer, EGFR can be activated through constitutive kinase activity triggered by somatic mutations (Zhou et al., 2024), which can continuously activate the EGFR signaling pathway and promote uncontrolled cell proliferation. Previous studies have demonstrated high expression of EGFR in breast cancer, and EGFR activation or overexpression is associated with metastasis (Zou et al., 2023). This study confirmed the high expression of EGFR at both transcriptional and protein levels in breast cancer tissues through the integration of bioinformatics analysis, tissue microarray validation, and molecular biology detection (Western blot/RT-qPCR) (Figure 1). The study’s use of only 14 clinical samples to evaluate EGFR expression limits the generalizability and reliability of the findings. The small sample size hinders representing the heterogeneity among breast cancer patients and may introduce bias in analyzing the relationship between EGFR expression and clinic pathological characteristics. Insufficient sample size also impedes subgroup analyses, affecting the accurate identification of EGFR expression patterns across different breast cancer subtypes. Furthermore, due to the limited sample size, the study did not conduct survival analysis or multivariate regression analysis to assess EGFR’s clinical value as a potential biomarker.

TNBC, comprising only 15%-20% of breast cancer cases, exhibits limited therapeutic targets and aggressive characteristics, resulting in a poorer prognosis compared to other subtypes. Due to often being identified in advanced stages, TNBC presents a heightened risk of recurrence and lower survival rates for patients (Garrido-Castro et al., 2019). Tumor metastasis is the most complex and clinically challenging biological process during cancer progression (Yan et al., 2025). Breast cancer invasion and metastasis entail a complex process, involving numerous genes and related signaling pathways. Among them, EMT is regarded as a necessary process for most cancers to metastasize (Williams et al., 2019). EMT, serving as the initiating link in the metastatic cascade, remodels cellular phenotypes by down-regulating epithelial markers (such as E-cadherin) and up-regulating mesenchymal markers (including vimentin and N-cadherin), endowing tumor cells with the ability of migration and invasion. To clarify EGFR’s regulatory role in EMT, this study constructed vectors with constitutively activated (Del19 + L858R mutation) and overexpressed EGFR. Results showed that EGFR activation or overexpression could significantly upregulate the expression of mesenchymal markers of EMT (vimentin, N-cadherin), while inhibiting the expression of epithelial marker (E-cadherin). This aligns with previous research findings. EGFR activation or overexpression can induce the EMT process of breast cancer cells (Garrido-Castro et al., 2019; Parker et al., 2023). Previous research has reported a correlation between abnormal EGFR expression and the migratory and invasive behavior of breast cancer cells (Williams et al., 2019). Results of the cell scratch assay in this study also showed that EGFR activation or overexpression could significantly enhance the migration ability of breast cancer cells (Figure 2). In conclusion, epithelial cells of breast cancer may induce EGFR activation or overexpression to trigger EMT and invasion and migration.

EGFR belongs to the receptor tyrosine kinase family. In this study, we used bioinformatics approaches to analyze receptors associated with EGFR and rank them based on correlation. We selected the top 6 receptors for further research. Through agonist stimulation experiments, we found that EGFR only activated by EGF. It is widely recognized that, for canonical EGFR signaling pathways, EGFR requires ligand binding to form an asymmetric dimer, thereby initiating its kinase activity. Natural gel electrophoresis detection revealed that EGFR activation, along with its facilitation of breast cancer EMT and invasion-metastasis, may be linked to receptor polymerization. Furthermore, treatment with Regorafenib significantly decreased the activity of downstream signaling pathways of EGFR, including Src-JNK/p38 pathway, and NF-κB-STAT3 pathway, and increased Hippo-YAP1 pathway. Previous studies have demonstrated that (i) Src is a key upstream regulator of YAP1 activation, influencing cell rearrangement and angiogenic growth (Lee et al., 2025); (ii) the Src/YAP1 axis is a well-characterized signaling pathway that facilitates cancer metastasis (Duan et al., 2024; Zhang et al., 2025); and (iii) EGFR has been shown to interact with the Src family kinases in previous reports (Fang et al., 2021; Chen et al., 2018). These references further support our proposed mechanism.

We also propose that the primary explanation for these discrepancies is that Regorafenib regulates MMP1 at the post-transcriptional level. To support this, we conducted several experiments. First, we performed time-course experiments to measure the mRNA and protein levels of MMP1 in Regorafenib-treated MCF-7 cells at various time points (0h, 3h, 6h, 12h, 24h, 36h, and 48h). The results indicated a significant downregulation of mRNA levels at 6–12 h, while protein levels remained stable throughout the time course (Supplementary Figure S4). This suggests that transcriptional inhibition is a transient effect that does not disrupt protein homeostasis.

This finding indicates that Regorafenib may impede breast cancer EMT, invasion, and metastasis by suppressing downstream of EGFR signal pathways. A potential limitation of pharmacological inhibitors is their risk of off-target effects. To mitigate this concern and reinforce our conclusions, this study combined pharmacological inhibition with Regorafenib and targeted EGFR knockdown through RNA interference. The consistent phenotypic changes observed with both methods provide strong evidence that the effects are specifically mediated by EGFR, rather than by non-specific actions of Regorafenib. This dual validation strategy enhances the causal relationship between EGFR signaling and tumor cell migration in our model system. Moreover, Regorafenib inhibited breast cancer metastasis might be related with glucose metabolism and the specific mechanism still needs further in-depth study.

## 5 Conclusion

In conclusion, our discovery of Regorafenib as a novel inhibitor of EGFR activation provides valuable insights for TNBC-specific anti-EGFR therapies targeting metastasis.

## Data Availability

The datasets presented in this study can be found in online repositories. The names of the repository/repositories and accession number(s) can be found in the article/Supplementary Material.
