# Supplementary material for: A novel kinase inhibitor, Regorafenib, blocks EGFR-dependent signaling to repress tumour metastasis in human triple-negative breast cancers
Source: Front Cell Dev Biol. 2026 Mar 3;14:1714597. doi: 10.3389/fcell.2026.1714597 (PMC12992224; doi:10.3389/fcell.2026.1714597)
Supplement: Supplementary file 1 [file DataSheet1.docx]

**Supplementary data**

**Fig. S1**

**Fig. S2**

**Table.1**

Q-PCR primers

| Name | Sequence (5’→3’) |
| --- | --- |
| N-cadherin-F | TTTGATGGAGGTCTCCTAACACC |
| N-cadherin-R | ACGTTTAACACGTTGGAAATGTG |
| E-cadherin-F | CGAGAGCTACACGTTCACGG |
| E-cadherin-R | GGGTGTCGAGGGAAAAATAGG |
| Vimentin-F | GCCCTAGACGAACTGGGTC |
| Vimentin-R | GGCTGCAACTGCCTAATGAG |
| EGFR-F | CCCACTCATGCTCTACAACCC |
| EGFR-R | TCGCACTTCTTACACTTGCGG |
| PNKP-F | CGAAACCAAGTGGAGCTGATT |
| PNKP-R | CAGTCCTGGCTTCAACTCCTG |
| MMP1-F | TGTTTGCAGAGCACTACTTGAA |
| MMP1-R | CAGTCACCTCTAAGCCAAAGAAA |
| MMP2-F | ACCTGAACACTTTCTATGGCTG |
| MMP2-R | CTTCCGCATGGTCTCGATG |
